# Supplementary figures and images for: APOBEC3 degradation is the primary function of HIV-1 Vif determining virion infectivity in the myeloid cell line THP-1
Source: mBio. 2023 Aug 9;14(4):e00782-23. doi: 10.1128/mbio.00782-23 (PMC10470580; doi:10.1128/mbio.00782-23)

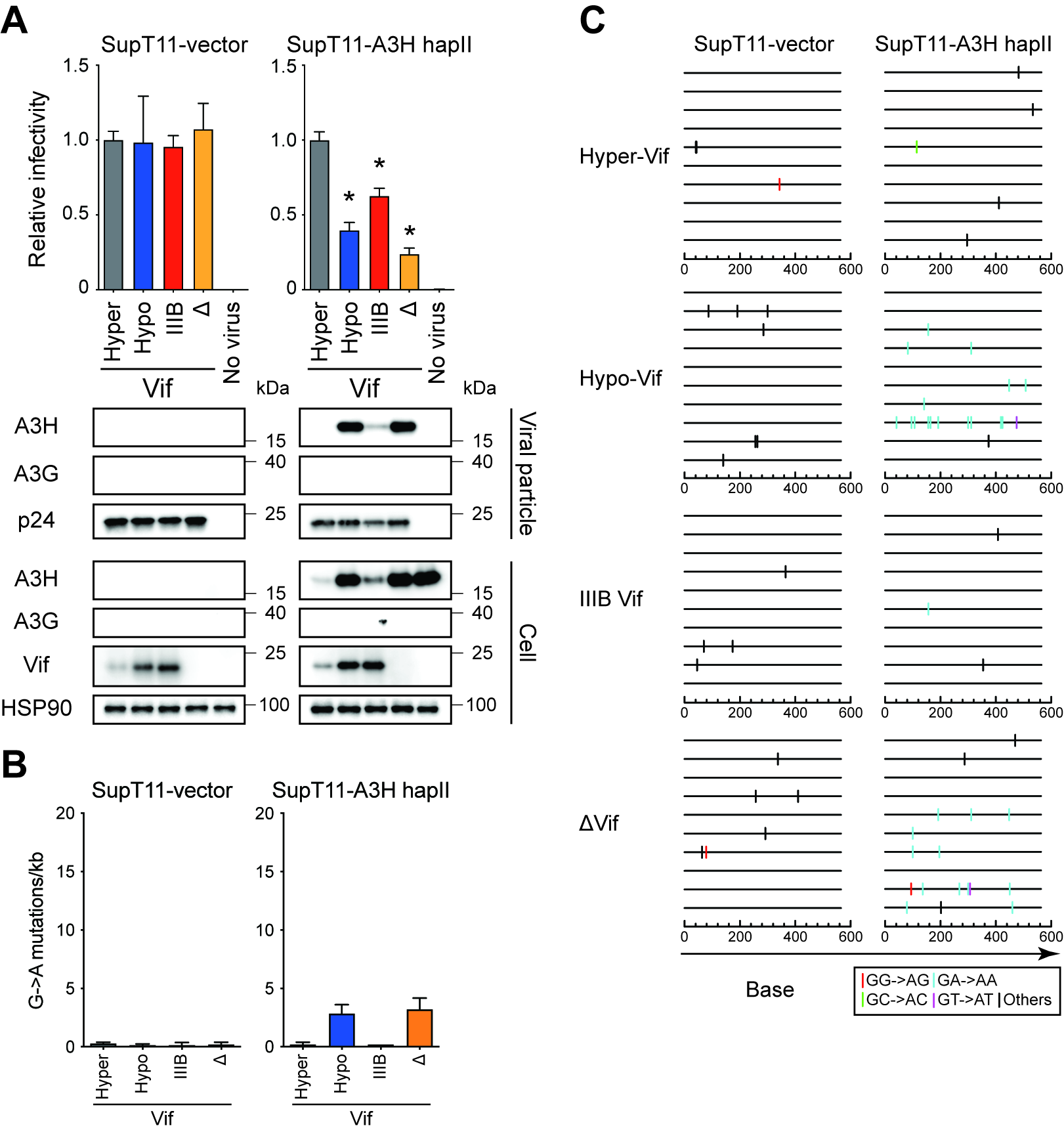

Supplement: Figure S1 — Pseudo-single cycle infectivity assays for each HIV-1 mutant in SupT11 cells stably expressing stable A3H haplotype. [file mbio.00782-23-s0001.tif]

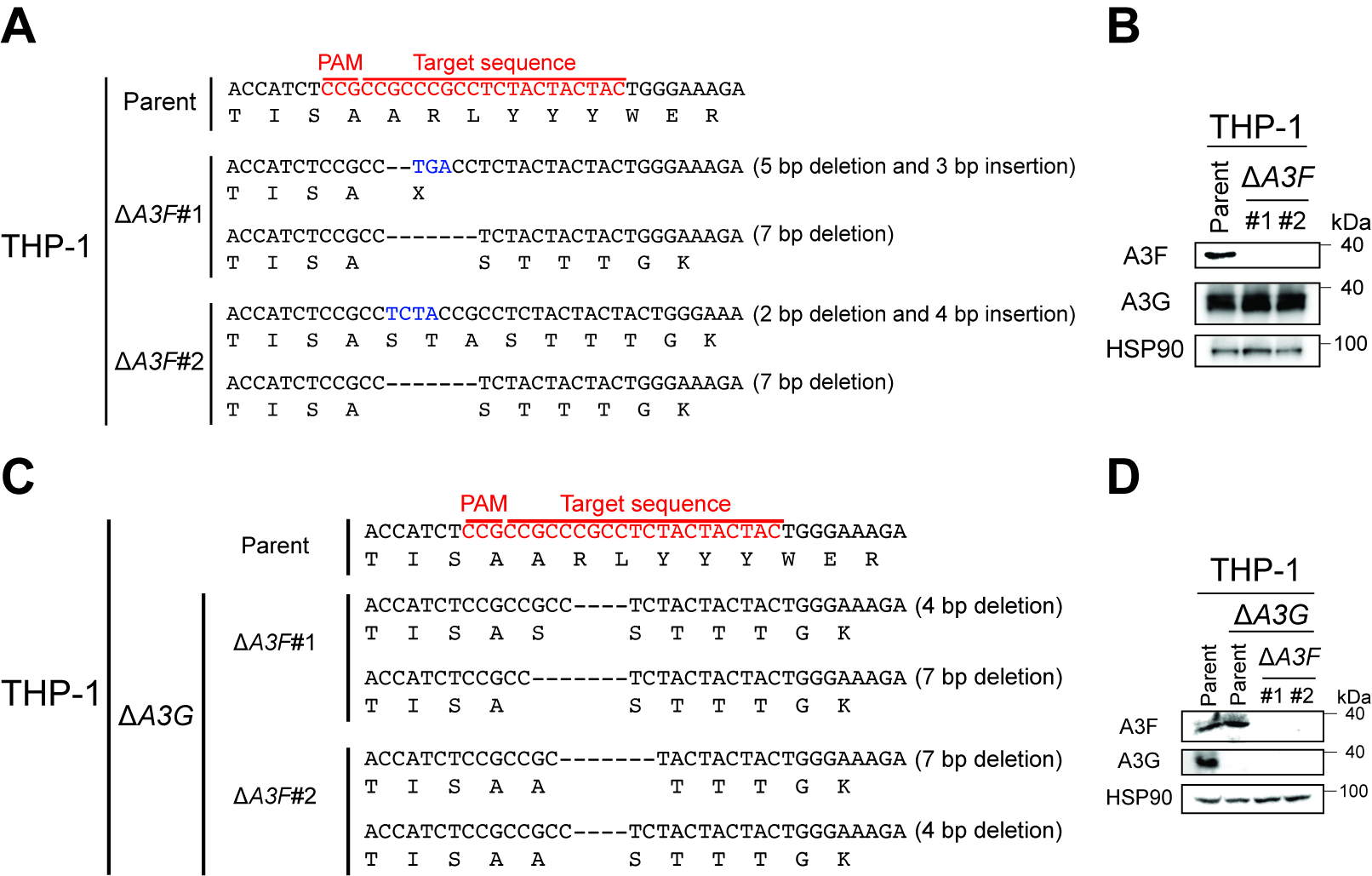

Supplement: Figure S2 — Development of A3F and A3F/A3G-null THP-1 cells. [file mbio.00782-23-s0003.tif]

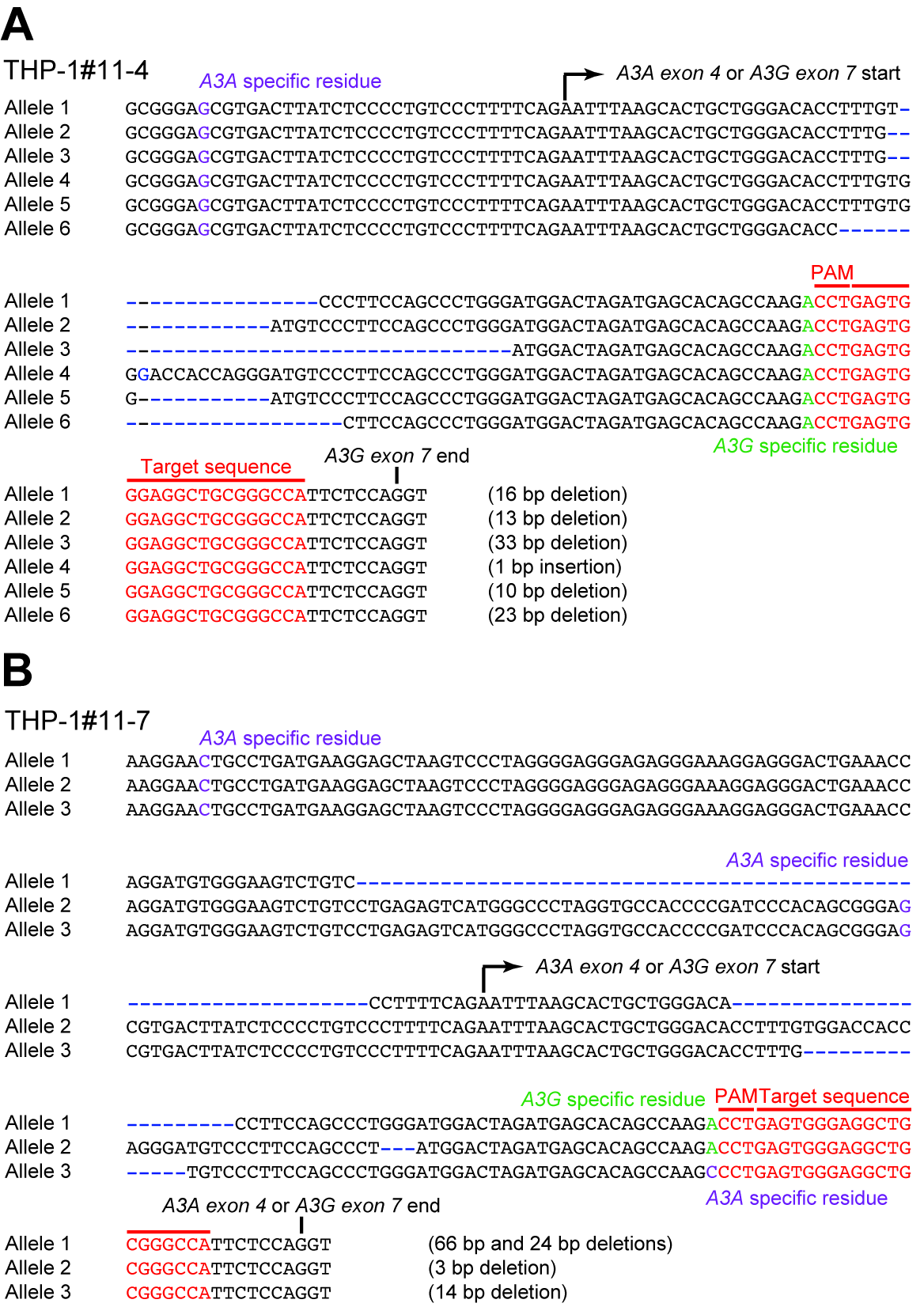

Supplement: Figure S3 — Sequence analysis of flanking region targeted by gRNA in THP-1#11-4 and #11-7. [file mbio.00782-23-s0004.tif]

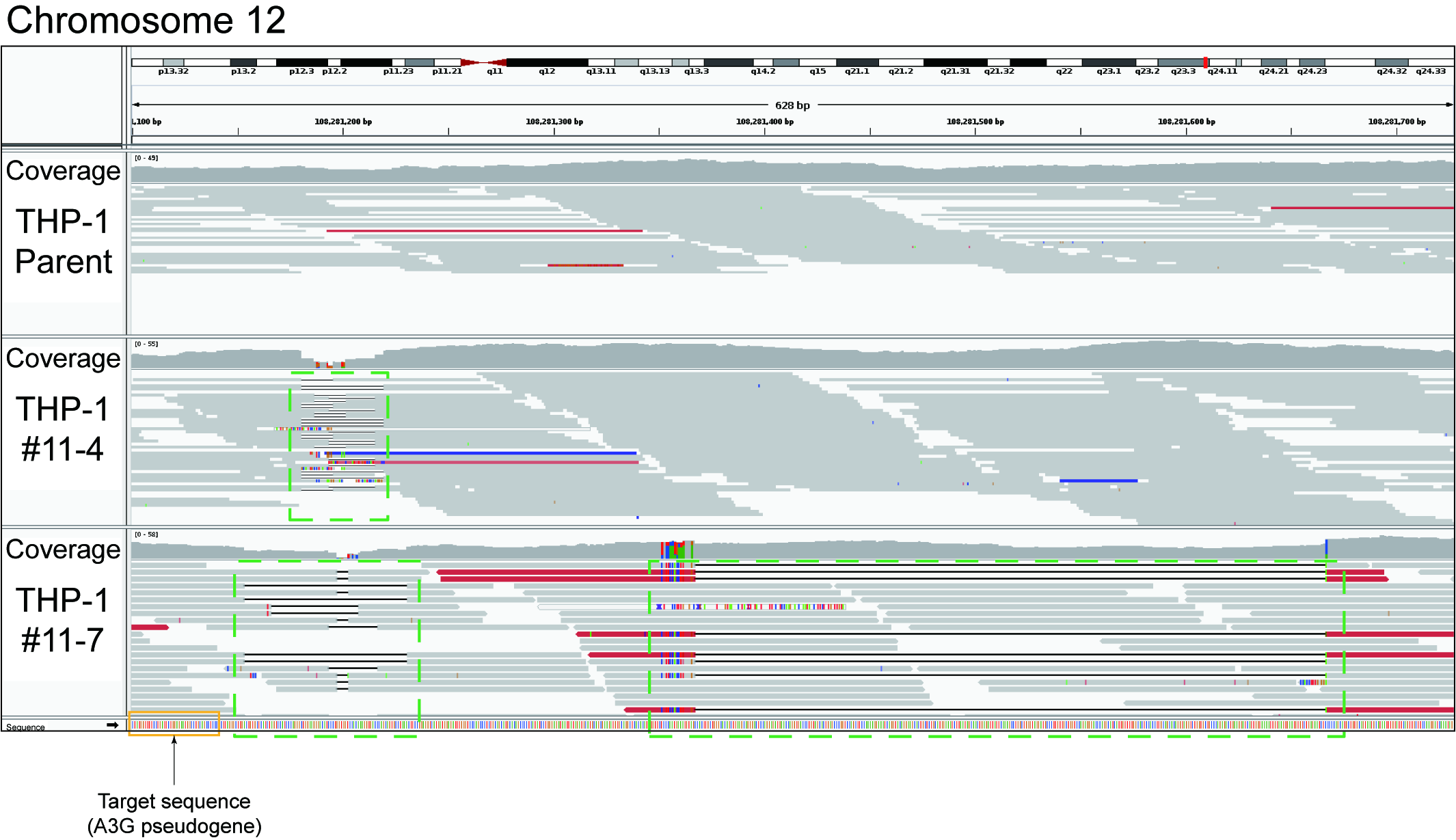

Supplement: Figure S4 — Deletions around predicted A3G pseudogene. [file mbio.00782-23-s0005.tif]

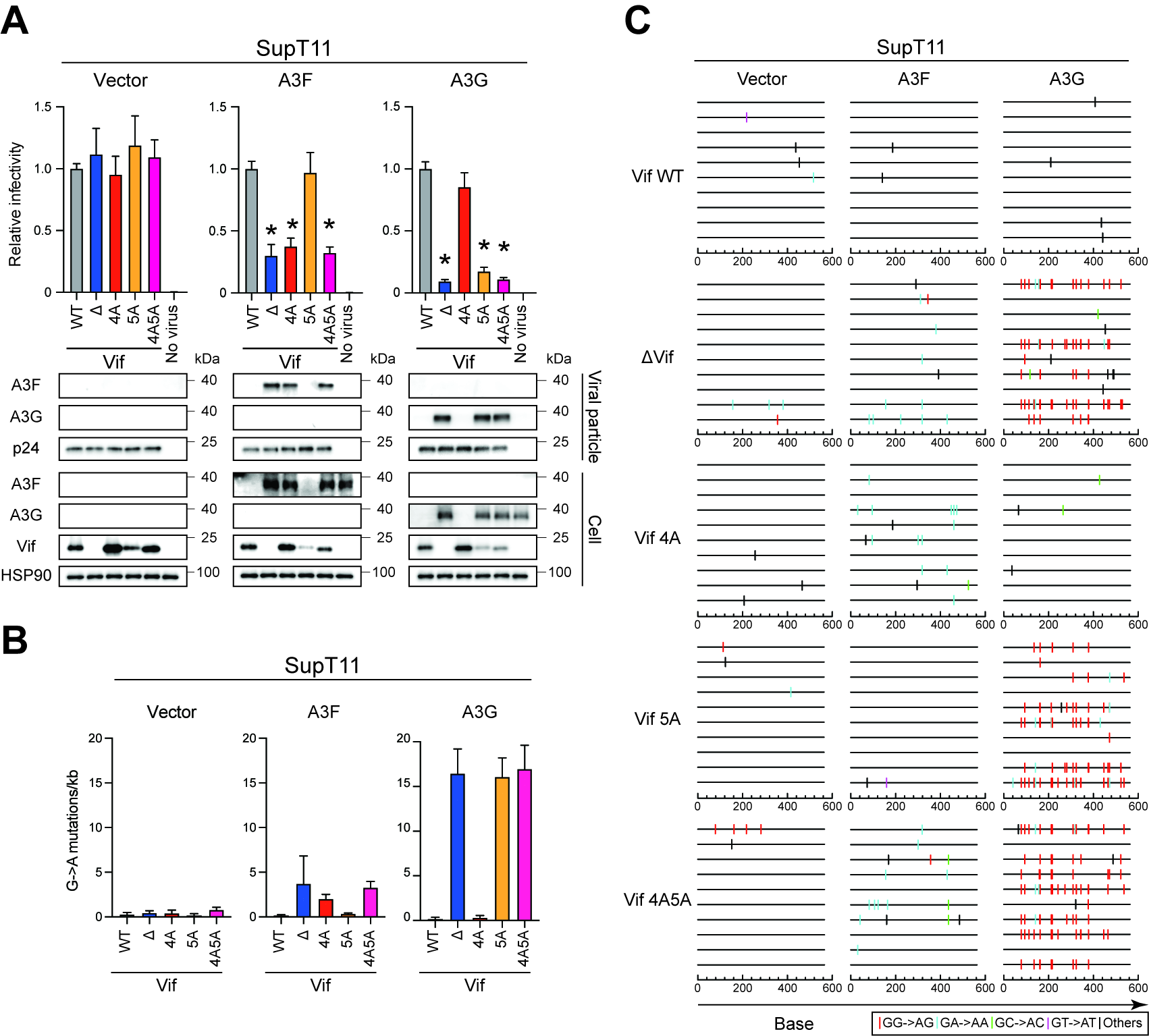

Supplement: Figure S5 — Pseudo-single cycle infectivity assays for each HIV-1 mutant in SupT11 cells stably expressing A3. [file mbio.00782-23-s0006.tif]
