# Supplementary material for: APOBEC3 degradation is the primary function of HIV-1 Vif determining virion infectivity in the myeloid cell line THP-1
Source: mBio. 2023 Aug 9;14(4):e00782-23. doi: 10.1128/mbio.00782-23 (PMC10470580; doi:10.1128/mbio.00782-23)
Supplement: Supplemental figure legends — Legends to Fig. S1 to S5. [file mbio.00782-23-s0002.docx]

APOBEC3 degradation is the primary function of HIV-1 Vif determining virion infectivity in the myeloid cell line THP-1

Terumasa Ikeda, Ryo Shimizu, Hesham Nasser, Michael A. Carpenter, Adam Z. Cheng, William L. Brown, Daniel Sauter, Reuben S. Harris

Supplemental figure legends for:

Figure S1

Figure S2

Figure S3

Figure S4

Figure S5

**Figure S1. Pseudo-single cycle infectivity assays for each HIV-1 mutant in SupT11 cells stably expressing stable A3H haplotype.**

(**A**) Representative infectivity of hyper- and hypo-functional Vif HIV-1 mutants produced from SupT11 cells stably expressing vector control and A3H hapII. Top panels show the infectivity of hyper-Vif, hypo-Vif, and IIIB Vif, and Vif-deficient HIV-1 mutants produced in SupT11 cells with stable expression of the control vector or A3H hapII. The amounts of produced viruses used to infect TZM-bl cells was normalized to p24 levels. Each bar shows the average of four independent experiments with SD. Data are represented as relative infectivity compared to hyper-Vif HIV-1. Statistical significance was determined using the two-sided paired *t* test. *P < 0.05 compared with the infectivity of hyper-Vif HIV-1. The bottom panels are representative western blots of three independent experiments. The levels of viral and cellular proteins in viral particles and whole cell lysates are shown. p24 and HSP90 were used as loading controls.

(**B**) G-to-A mutations. Average number of G-to-A mutations in the 564 bp *pol* gene after infection with hyper-Vif, hypo-Vif, IIIB Vif, or Vif deficient HIV-1 produced from SupT11 cells expressing either the vector control or A3H hapII. Each bar depicts the average of three independent experiments with SD.

(**C**) G-to-A mutation profile. Dinucleotide sequence contexts of G-to-A mutations in the 564 bp *pol* gene after infection with the indicated viruses produced from indicated cell lines. Each vertical line indicates the location of the dinucleotide sequence contexts described in the legend within the 564 bp amplicon (horizontal line).

**Figure S2. Development of *A3F* and *A3F/A3G*-null THP-1 cells.**

(**A**) *A3F* exon 3 sequences encompassing the gRNA target site in parental THP-1 and two independent *A3F*-null THP-1 cells. Indels in two alleles for each *A3F*-null THP-1 clone are shown.

(**B**) Representative western blots of three independent experiments. Levels of A3F and A3G protein in whole cell lysates are shown. HSP90 was used as a loading control.

(**C**) *A3F* exon 3 sequences encompassing the gRNA target site in parental THP-1 and two independent *A3F/A3G*-null THP-1 cells. Indels in two alleles for each *A3F/A3G*-null THP-1 clone are shown.

(**D**) Representative western blots of three independent experiments. Levels of A3F and A3G protein in whole cell lysates are shown. HSP90 was used as a loading control.

**Figure S3. Sequence analysis of flanking region targeted by gRNA in THP-1#11-4 and #11-7.**

(**A**) *A3A* exon 4 and *A3G* exon 7 hybrid sequences encompassing the gRNA target site in THP-1#11-4 cells. Only one nucleotide difference (>99% identity) was observed between *A3A* exon 4 and *A3G* exon 7 and is shown in purple (A3A, cytosine) or green (A3G, adenine). Indels in six alleles of the THP-1#11-4 clone are shown.

(**B**) *A3A* exon 4 and *A3G* exon 7 hybrid sequences encompassing the gRNA target site in THP-1#11-7 cells. Only one nucleotide difference (>99% identity) was observed between *A3A* exon 4 and *A3G* exon 7 and is shown in purple (A3A, cytosine) or green (A3G, adenine). Indels in three alleles of the THP-1#11-7 clone are shown.

**Figure. S4. Deletions around predicted *A3G* pseudogene.**

Mapping of WGS sequencing data to off-target and downstream regions on chromosome 12. Genomic DNA from parental THP-1, THP-1#11-4, and THP-1#11-7 cells were subjected to WGS analysis. The orange box indicates the off-target sequence in the predicted pseudogene. Several deletions were observed in the regions indicated by green dot boxes in THP-1#11-4 and THP-1#11-7 clones.

**Figure S5. Pseudo-single cycle infectivity assays for each HIV-1 mutant in SupT11 cells stably expressing A3.**

(**A**) Representative infectivity of Vif-proficient, Vif-deficient, Vif4A, Vif5A, and Vif4A5A HIV-1 mutants produced from SupT11 cells stably expressing vector control, A3F, or A3G. Top panels show the infectivity of indicated HIV-1 mutants produced in SupT11 cells stably expressing either vector control, A3F, or A3G. The amounts of produced viruses used to infect TZM-bl cells was normalized to p24 levels. Each bar represents the average of four independent experiments with SD. Data are presented as relative infectivity compared to Vif-proficient HIV-1 (WT). Statistical significance was assessed using the two-sided paired *t* test. *P < 0.05 compared to Vif-proficient HIV-1. Bottom panels are representative western blots of three independent experiments. Levels of indicated viral and cellular proteins in viral particles and whole cell lysates are shown. p24 and HSP90 were used as loading controls.

(**B**) G-to-A mutations. Average number of G-to-A mutations in the 564 bp *pol* gene after infection with Vif-proficient, Vif-deficient, Vif4A, Vif5A, and Vif4A5A HIV-1 mutants produced from SupT11 cells stably expressing either vector control, A3F, or A3G. Each bar depicts the average of three independent experiments with SD.

(**C**) G-to-A mutation profile. Dinucleotide sequence contexts of G-to-A mutations in the 564 bp *pol* gene after infection with the indicated viruses produced from indicated cell lines. Each vertical line indicates the location of the dinucleotide sequence contexts described in the legend within the 564 bp amplicon (horizontal line).
